# Comparative physiology of allopatric *Populus* species: geographic clines in photosynthesis, height growth, and carbon isotope discrimination in common gardens

**DOI:** 10.3389/fpls.2015.00528

**Published:** 2015-07-14

**Authors:** Raju Y. Soolanayakanahally, Robert D. Guy, Nathaniel R. Street, Kathryn M. Robinson, Salim N. Silim, Benedicte R. Albrectsen, Stefan Jansson

**Affiliations:** ^1^Agroforestry Development Centre, Agriculture and Agri-Food CanadaIndian Head, SK, Canada; ^2^Department of Forest and Conservation Sciences, University of British ColumbiaVancouver, BC, Canada; ^3^Department of Plant Physiology, Umeå Plant Science Centre, Umeå UniversityUmeå, Sweden

**Keywords:** comparative physiology, poplar, common garden, latitude, bud set, photosynthesis, carbon isotope discrimination, water-use efficiency

## Abstract

*Populus* species with wide geographic ranges display strong adaptation to local environments. We studied the clinal patterns in phenology and ecophysiology in allopatric *Populus* species adapted to similar environments on different continents under common garden settings. As a result of climatic adaptation, both *Populus tremula* L. and *Populus balsamifera* L. display latitudinal clines in photosynthetic rates (*A*), whereby high-latitude trees of *P. tremula* had higher *A* compared to low-latitude trees and nearly so in *P. balsamifera* (*p* = 0.06). Stomatal conductance (*g*_s_) and chlorophyll content index (CCI) follow similar latitudinal trends. However, foliar nitrogen was positively correlated with latitude in *P. balsamifera* and negatively correlated in *P. tremula*. No significant trends in carbon isotope composition of the leaf tissue (δ^13^C) were observed for both species; but, intrinsic water-use efficiency (WUE_i_) was negatively correlated with the latitude of origin in *P. balsamifera*. In spite of intrinsically higher *A*, high-latitude trees in both common gardens accomplished less height gain as a result of early bud set. Thus, shoot biomass was determined by height elongation duration (HED), which was well approximated by the number of days available for free growth between bud flush and bud set. We highlight the shortcoming of unreplicated outdoor common gardens for tree improvement and the crucial role of photoperiod in limiting height growth, further complicating interpretation of other secondary effects.

## Introduction

The “common garden” approach (Kawecki and Ebert, 2004), whereby samples of genotypes collected from different populations are directly compared under the same environmental conditions, either outdoors in a greenhouse, or in a growth chamber, is widely used to study local adaptation. Since the late 18th century, field “provenance trials” have been used in forestry to ensure that sources of seed produce well-adapted, productive trees for reforestation or afforestation (Linhart and Grant, 1996). Common garden experiments or provenance trials also provide material for tree breeding and *ex-situ* conservation. Through association studies, common gardens that include accessions from a large number of populations have become extremely useful in uncovering the molecular-genetic basis for differences in physiological traits and their functional regulation (McKown et al., 2013a, 2014). They also yield invaluable data to model and predict tree responses to climate change (Wang et al., 2006), especially when replicated across the landscape (e.g., Rehfeldt et al., 1999).

Phenotypic selection studies, including those that describe the relationships of plant physiological traits across latitudes, can be used to examine the evolution of particular traits and to test adaptive hypotheses. Variations in growth rates and morphological characters have been well documented in latitudinally separated populations that have been sampled or collected over vast geographic ranges. In this context, latitude is really a proxy for environmental factors that drive selection, such as growing season length, photoperiod, temperature, and water availability. The general understanding is that high-latitude plants grow more slowly and are shorter in height compared to plants from low latitudes (Luquez et al., 2007; Moles et al., 2009). Differences in stature, however, may depend more on phenology and environment than on intrinsic growth potential. Indeed, high latitude populations of balsam poplar (*Populus balsamifera* L., section: *Tacamahaca*) are capable of higher rates of carbon assimilation (*A*) and height growth than their southern counterparts when freed from photoperiodic restrictions (Soolanayakanahally et al., 2009). The timing of height growth cessation is inversely correlated with the latitude of origin, and photoperiodic responses may be the only major adaptive mechanism responsible for this phenomenon (Schnekenburger and Farmer, 1989). Quite similarly, Howe et al. (1995) observed a longer critical photoperiod for bud set in *P. trichocarpa* ecotypes from 53°N compared to ecotypes from 34°N.

Ecophysiological attributes such as photosynthesis, respiration, leaf nitrogen (N), and growth phenology all vary with leaf lifespan (Wright et al., 2004a). Trait correlations across major plant functional groups strongly associate leaf lifespan and leaf mass per area (LMA) with climate variation (Wright et al., 2004b). Species with short-lived foliage generally have higher *A*, owing to trade-offs in carbon and nitrogen allocation between biochemistry and structure. A strong positive correlation between photosynthesis of a leaf and its nitrogen concentration is well recognized in plant species (Evans, 1989). Even in deciduous trees, where the green-cover period (GCP) is less than 1 year, there is a negative relationship between *A* and leaf lifespan among (Reich et al., 1995) and within (e.g., Gornall and Guy, 2007) species. Classic studies of adaptive variation in *Oxyria digyna* (Mooney and Billings, 1961; Billings et al., 1971) indicated a latitudinal cline in photosynthesis toward higher *A* in arctic ecotypes. Particularly in *Populus* species occupying large geographic areas, enhanced photosynthetic rates are driven by larger stomatal conductance (*g*_s_) (Gornall and Guy, 2007; Soolanayakanahally et al., 2009; McKown et al., 2013a). The existence of such clines, especially when common to more than one species along the same or similar environmental gradient, constitutes strong evidence for adaptive trait selection (Endler, 1973).

Landscape patterns in ecophysiological traits of vegetation are beginning to be recognized at species and community levels, but rarely within species, which can be at odds with higher levels of organization. For example, Soolanayakanahally et al. (2009) reported that LMA increases with latitude of origin within balsam poplar, whereas in a meta-analysis of data for 2548 species, Wright et al. (2004b) found the opposite trend across biomes. In this study, we examine latitudinal patterns in photosynthesis as an adaptation to growing season length in allopatric species of *Populus*. The genus *Populus* presents many advantages to comparative physiologists seeking to understand the biological significance of physiological adaptation, including ease of propagation, access to large collections of native germplasm from temperate through sub-arctic habitats, and substantial genetic tools and resources.

Stomata partly determine intrinsic water-use efficiency (WUE_i_), which is important to plant productivity, by mediating the diffusion of CO_2_ into leaves and water vapor out. Assimilation-averaged water-use efficiency can be estimated *via* its correlation with the stable carbon isotopic composition of plant tissues (Farquhar et al., 1982). The carbon isotope ratio (δ^13^C) of plant tissue provides an integrated measure of internal plant physiological and external environmental properties influencing photosynthesis over the time when the carbon was fixed (Anderson et al., 1996). Variations in WUE_i_ and δ^13^C have been well characterized along geographic gradients in moisture, temperature, and nutrients in many species. The variations in δ^13^C have adaptive significance in *Pinus ponderosa* Dougl. *ex* Laws. (Zhang et al., 1997), *Chrysothamnus nauseosus* (Donovan and Ehleringer, 1994), *Pinus contorta* Dougl. *ex* Loud. (Guy and Holowachuk, 2001), and *Populus nigra* L. (Chamaillard et al., 2011).

Although species of different origin can display similar clines in phenotypic traits as a result of natural selection, a preferred phenotype may be achieved by different combinations of underlying traits. For example, latitudinal variation in *A* is associated with higher *g*_s_ among field-grown populations of black cottonwood (*Populus trichocarpa* Torr. & Gray, section: *Tacamahaca*) (Gornall and Guy, 2007), but not in greenhouse-grown balsam poplar (Soolanayakanahally et al., 2009), with consequent effects on respective trends in WUE_i_ and δ^13^C. This is somewhat surprising given that these two North American species are very closely related and hybridize extensively where their ranges overlap (Farrar, 1995). We wondered, therefore, whether similar patterns in *A*, *g*_*s*_, and related ecophysiological traits would be found in a more disparate member of the genus *Populus*. In this study, we compared data of different *Populus* species, grown under natural conditions in common gardens in the field, to understand commonalities and differences within the genus. Relevant data were already available for *P. trichocarpa*, so we generated similar datasets for *P. tremula* and *P. balsamifera*. We then used the combined dataset to answer the following questions:

1. Do latitudinal clines in *A* exist in allopatric *Populus* species adapted to similar environments on different continents when measured during active growth in common gardens?
2. Are clinal patterns in height growth better explained by timing of bud set or by photosynthetic assimilation rates?

## Material and methods

### Balsam poplar common garden

Balsam poplar has a continuous natural range from Alaska to Newfoundland and south to Michigan. The plant material used here is a subset of the larger Agriculture Canada Balsam Poplar (AgCanBaP) collection consisting of 65 provenances (Soolanayakanahally et al., 2013). For the present purposes, five populations (Table 1) with 15 genotypes per population were planted into an outdoor common garden at Indian Head (50.33° N 103.39° W), Canada in mid-August 2005. This location is near the southern edge of the species range where photoperiod is always limiting during summer for most of the high-latitude populations. Stem cuttings (6–9 cm in length) with a minimum of two buds were rooted in Spencer-Lemaire RT 420A Rootrainer® containers (Beaver Plastics, Acheson, Canada) filled with a mixture of Sunshine-2 (Sun Gro Horticulture, Vancouver, Canada) growing mix (60%), peat (30%), and vermiculite (10%). The rooted cuttings were grown in a greenhouse with natural light supplemented by cool-white fluorescent lamps to provide a 19-h photoperiod and a minimum PPFD of 400 μmol m^−2^ s^−1^ at plant level. Maximum day and night temperatures were maintained close to 25 and 18°C, respectively. Upon flushing, the cuttings were kept well watered and fertilized weekly with ½-strength Hoagland's solution (Hoagland and Arnon, 1950). When the plants were approximately 45 cm tall, they were moved to a shade house for a period of 2 weeks before planting in a complete block design with three replicates per genotype spaced two meters apart. At the common garden site, maximal photoperiod is 16 h 12 min, with mean annual precipitation of 435 mm, and mean maximum and minimum temperatures during the growing season (May–August) of 22 and 8°C, respectively. The year in which measurements were taken, 2007, was relatively wet. The garden was weeded annually.

**Table 1:** **Geographic coordinates and mean elevation of origin for populations established in common gardens, by species**.

**Species**	**Population**	**Latitude**	**Longitude**	**Elevation**
*P. balsamifera* (North America)	Fredericton (FRE)	46.40	67.25	147
	Rouyn Noranda (RNA)	48.60	78.67	310
	Love (LOV)	53.63	105.50	419
	Grand Prairie (GPR)	54.75	118.63	769
	White Horse (WHR)	60.70	135.33	770
*P. tremula* (Europe)	Ronneby	56.27	15.21	49
	Simlång	56.71	13.25	173
	Ydre	57.79	15.28	219
	Vårgårda	57.99	12.93	158
	Brunsberg	59.63	12.96	84
	Uppsala	59.81	17.91	17
	Älvdalen	61.22	13.97	354
	Delsbo	61.73	16.71	98
	Umeå	63.93	20.63	37
	Dorotea	64.36	16.44	382
	Luleå	65.62	22.19	13
	Arjeplog	66.20	18.43	445

*Latitude ( ° N), longitude ( ° W, North America and ° E, Europe), elevation (m)*.

### Aspen common garden

Eurasian aspen is distributed throughout Europe from the Mediterranean to northern Scandinavia and eastwards to Siberia and central Asia. We used the Swedish Aspen (SwAsp) collection planted at Sävar (63.80° N 20.30° E) in June 2004. The SwAsp collection contains 116 genotypes collected from 12 localities in Sweden (Table 1, 10 genotypes per population except for Luleå where only six genotypes were sampled). For more information on the collection and establishment of the common garden, refer to Luquez et al. (2007). In contrast to balsam poplar, the aspen common garden is located close to the northern edge of the species range, and photoperiod is not limiting during summer. At Sävar, the maximal photoperiod is 20 h 53 min, with mean annual precipitation of 482 mm, and mean maximum and minimum temperatures during the growing season (May–August) of 23 and 6°C, respectively. Spot weeding was done annually within a 0.5 m perimeter around each tree.

### Gas exchange measurements

In both species, measurements were taken during active growth (i.e., well before bud set) in the absence of any water stress. For balsam poplar, measurements were made in the first 2 weeks of July 2007 on five populations (six trees per population; *n* = 30). For aspen, measurements were made on all 116 genotypes from June 24 to July 10 2008. Gas exchange was measured on all trees once, with the measurement tree randomized among populations and days of measurement.

Carbon assimilation (*A*), measured as light saturated photosynthesis, and stomatal conductance (*g*_s_) were measured on clear, sunny days using a LC *Pro*^+^ portable gas exchange system (Analytical Development Co., Ltd., Hoddesdon, UK). Measurements were made between 9:00 and 12:00 h on fully expanded sun leaves on each tree. CO_2_ concentrations inside the cuvette were 360–370 μL L^−1^. Air temperature was maintained at 25°C to yield a leaf temperature of approximately 26.5°C. Relative humidity was 60–70%, resulting in a leaf-to-air vapor pressure difference of 1.24–1.56 kPa. PPFD was 1500 μmol m^−2^ s^−1^ supplied by a mixed red/blue LED unit mounted on top of the cuvette. After stabilization of intercellular CO_2_ concentration (*C*_*i*_), three measurements were recorded over a period of 3 min and averaged to determine *A* (μmol CO_2_ m^−2^ s^−1^) and *g*_s_ (mol H_2_O m^−2^ s^−1^) following von Caemmerer and Farquhar (1981). The WUE_i_ was calculated as the ratio of *A* to *g*_s_ (i.e., μmol CO_2_ mol^−1^ H_2_O).

Following gas exchange measurements, the chlorophyll content index (CCI) was determined on five leaves per tree with an Opti-Sciences CCM-200 meter (Hudson, NH, USA). The CCM-200 uses calibrated light emitting diodes and receptors to calculate the CCI, which is defined as the ratio of light transmission through the leaf at 931 nm to that at 653 nm. The efficacy of CCI for rapid and non-destructive estimation of relative total chlorophyll or foliar nitrogen content is well-established for many forest tree species (van den Berg and Perkins, 2004).

### Leaf tissue sampling

Whole leaf samples were collected from 75 balsam poplar trees (i.e., 15 genotypes × 5 populations) and 348 aspen trees (i.e., 116 genotypes × 3 ramets/genotype) after the gas exchange measurements were completed. These were used for analysis of δ^13^C and nitrogen content (Leaf N, μmol N cm^−2^). Leaf tissue was dried to constant mass and then ground to fine powder. Homogenized subsamples of ~2.5 mg were packed in tin capsules and sent to the University of California at Davis Stable Isotope Facility for combustion and analysis by an online continuous flow dual analyzer coupled to an isotope ratio mass spectrometer (Europa Scientific Integra, Cheshire, England, UK). The δ^13^C value of the leaf tissue is reported in per mil (‰) units relative to the arbitrary standard Vienna Pee Dee Belemnite (VPDB):

$$\begin{array}{l} \delta^{13}C=[{\left({}^{13}C{/}^{12}C\right)}_{\text{Sample}}-{\left({}^{13}C{/}^{12}C\right)}_{\text{VPDB}}]/{\left({}^{13}C{/}^{12}C\right)}_{\text{VPDB}}\\ \;\times 1000 \end{array}$$

The overall, long-term sample preparation and analysis error between repeated analyses of the same ground tissue was less than ±0.11‰.

### Growth measurements

Final height of all genotypes was measured after bud set in 2007 for balsam poplar and 2008 for aspen. The 30 balsam poplar trees used for gas exchange measurements were harvested for shoot biomass determination at the end of the 2007 growing season. Numbers of leaves were counted for each tree and the leaf area was measured using a LI-3100 leaf area meter (LI-COR Biosciences, Lincoln, NE, USA). Dry mass was recorded after the stems and leaves were oven-dried to a constant weight. LMA was expressed as the leaf mass to area ratio.

### Seasonal phenology

In both species, spring and autumn phenology, characterized by dates of bud flush and bud set, respectively, were monitored twice weekly. The phenological stages and their characteristics are described in Soolanayakanahally et al. (2013). A scale of 0–10 was used to describe the different phenological stages, where 0 represented a spring stage where the buds were still closed and non-swollen, and 10 an autumn stage when there was complete leaf senescence. Height elongation duration (HED) was calculated as the number of days between bud flush and bud set. For balsam poplar, the length of the GCP, defined as the number of days from bud flush to when 80% of the leaves had abscised, was also recorded.

### Statistical analysis

Data analyses were conducted in SigmaStat version 2.03. Pearson's correlation coefficients (*r*) among physiology, growth, and geographic variables were calculated to determine the relationships among all variables across genotypes. Later, Bonferroni correction was applied to minimize the chances of making a Type I error. Multiple linear regressions were carried out on the data to select latitude as a common descriptive variable. The population means with standard errors are reported.

## Results

For both of the collections used in this study, latitude, longitude, and elevation of origin are confounded, particularly in balsam poplar (Table 1). Although we report correlation coefficients between physiological variables and all three geographic descriptors in Tables 2, 3, relationships with longitude and/or elevation were in every case not significant after latitude was accounted for in multiple linear regressions (not presented). In balsam poplar, the number of frost-free days (which would tend to include the independent effects of latitude and elevation on temperature and growing season length) is actually a marginally better predictor than latitude in several cases (also not presented). However, for comparison to the other data sets generated or used in this study, we focus on latitude.

**Table 2:** **Pearson's correlation coefficients among physiology, growth, and geographic variables in** ***P. balsamifera*** **populations**.

**Variable**	**Latitude**	**Longitude**	**Elevation**
*A*	0.853	0.865	0.825
*g*_s_	**0.928**	**0.894**	0.845
WUE_i_	**−0.944**	**−0.885**	−0.802
δ^13^C	−0.668	−0.590	−0.582
Leaf N	0.968^*^	0.964^*^	**0.884**
CCI	**0.940**	**0.944**	**0.924**
LMA	0.999^*^	0.985^*^	**0.911**
GCP	−0.852	−0.759	−0.645
HED	−0.989^*^	−0.962^*^	−0.860
Height	**−0.941**	**−0.879**	−0.782
Biomass	**−0.904**	**−0.895**	−0.802

*Significant correlations are in bold (p < 0.05) or followed by an asterisk (p < 0.01). A, assimilation rate (μmol CO_2_ m^−2^ s^−1^); g_s_, stomatal conductance (mol H_2_O m^−2^ s^−1^); WUE_i_, intrinsic water use efficiency (μmol CO_2_ mol^−1^ H_2_O); δ^13^C, carbon isotope composition of leaf (‰); Leaf N, leaf nitrogen density (μmol N cm^−2^); CCI, chlorophyll content index; LMA, leaf mass area (mg cm^−2^); GCP, green-cover period (days); HED, height elongation duration (days); Height (cm) and Biomass (g)*.

**Table 3:** **Pearson's correlation coefficients among physiology, growth, and geographic variables in P. tremula populations**.

**Variables**	**Latitude**	**Longitude**	**Elevation**
A	0.798^*^	0.709^*^	0.158
*g*_s_	0.712^*^	0.575	0.264
WUE_i_	−0.564	−0.402	−0.269
δ^13^C	−0.572	−0.262	−0.525
Leaf N	−0.869^*^	**−0.609**	−0.224
CCI	0.832^*^	**0.695**	0.365
LMA	−0.443	−0.346	−0.139
HED	−0.945^*^	**−0.664**	−0.338
Height	−0.905^*^	**−0.645**	−0.331

*Significant correlations are in bold (p < 0.05) or followed by an asterisk (p < 0.01). A, assimilation rate (μmol CO_2_ m^−2^ s^−1^); g_s_, stomatal conductance (mol H_2_O m^−2^ s^−1^); WUE_i_, intrinsic water use efficiency (μmol CO_2_ mol^−1^ H_2_O); δ^13^C, carbon isotope composition of leaf (‰); Leaf N, leaf nitrogen density (μmol N cm^−2^); CCI, chlorophyll content index; LMA, leaf mass area (mg cm^−2^); HED, height elongation duration (days) and Height (cm)*.

**Figure 1:**
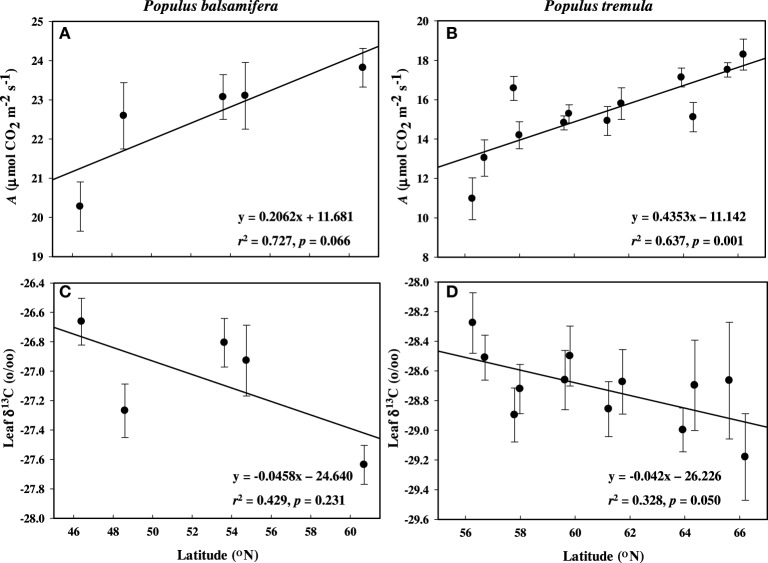
**Mean net assimilation rate (A) and leaf δ^13^C (‰) across latitude measured during active growth in common gardens**. (A,C) P. balsamifera and (B,D) P. tremula. Error bars are ±SE of the means.

During active growth (i.e., before bud set) field gas exchange measurements showed significantly higher A in aspen populations coming from high latitudes compared with those from low latitudes (*p* = 0.001; Figure 1B). However, there was a non-significant correlation between A and latitude in balsam poplar (*p* = 0.066; Figure 1A). Mean photosynthetic rate was higher in balsam poplar compared to aspen, but differences between populations (as a function of latitude of origin) were more pronounced in aspen. Variation in stomatal conductance (*g*_s_), as a function of latitude, paralleled variation in A in aspen (Tables 3) and the correlation between A and *g*_s_ was very strong for both P. balsamifera (*r* = 0.816) and P. tremula (*r* = 0.717).

WUE_i_ was negatively related with latitude in balsam poplar (*p* < 0.05, Table 2), however, δ^13^C was non-significant with latitude (p > 0.05, Table 2). In aspen, both WUE_i_ and δ^13^C were non-significant with latitude of origin (*p* > 0.05, Table 3). But, WUE_i_ and δ^13^C positively correlated with each other in balsam poplar (Supplement 1). Changes in δ^13^C as a function of latitude were similar in the two species (Figures 1C,D). In balsam poplar, WUE_i_ ranged from 57.9 to 69.2 μmol CO_2_ mol^−1^ H_2_O among populations, whereas in aspen it was from 43.3 to 59.1 μmol CO_2_ mol^−1^ H_2_O. The species difference in WUE_i_ was also reflected in δ^13^C values, which ranged from −26.66 to −27.64‰ in balsam poplar and −28.51 to −29.29‰ in aspen. Trees with more negative δ^13^C values (balsam poplar) or lower WUE_i_ (balsam poplar and aspen) had higher *g*_s_ (Supplement 1).

**Figure 2:**
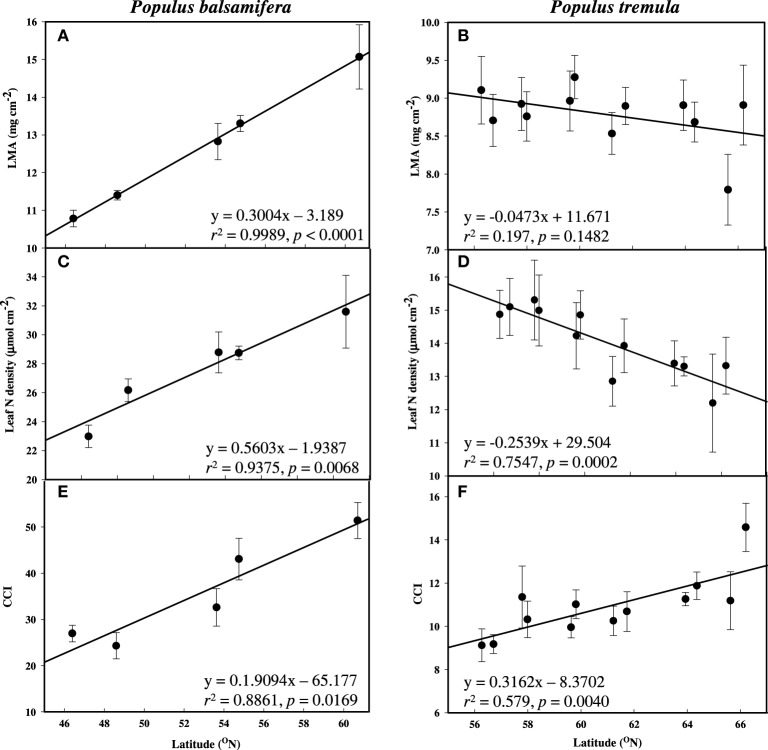
**Mean leaf mass area (LMA), leaf nitrogen density (Leaf N) and chlorophyll content index (CCI) across latitude measured during active growth in common gardens**. (A,C,E) P. balsamifera and (B,D,F) P. tremula. Error bars are ±SE of the means.

Leaf N increased significantly with latitude, longitude, and elevation in balsam poplar (Table 2 and Figure 2C). Both Leaf N and LMA were strongly positively correlated with all three geographic parameters. Nitrogen per unit mass was quite uniform (not presented). Therefore, enhanced A with latitude was associated with increased Leaf N more so than *g*_s_. The overriding influence of *g*_s_ was even more apparent in aspen, where Leaf N decreased significantly with latitude (Table 3, Figure 2D), while A was enhanced, again consistent with a reduced water-use efficiency. In sharp contrast to balsam poplar (Figure 2A), however, LMA in aspen had no correlation with latitude when calculated across all 116 genotypes (not presented) or across the 12 population means (Table 3, Figure 2B). Luleå, a northern population in Sweden, seemed to have an anomalously low LMA. Leaf N density was positively correlated with δ^13^C in aspen genotypes, but not with δ^13^C in balsam poplar (Supplement 1).

Although higher rates of A were not associated with higher Leaf N in aspen (in contrast to balsam poplar), they were supported by higher chlorophyll per unit leaf area, as indicated by CCI. CCI was positively and significantly related to latitude (Figures 2E,F) and longitude in both species, and most particularly in balsam poplar (Tables 2, 3).

**Figure 3:**
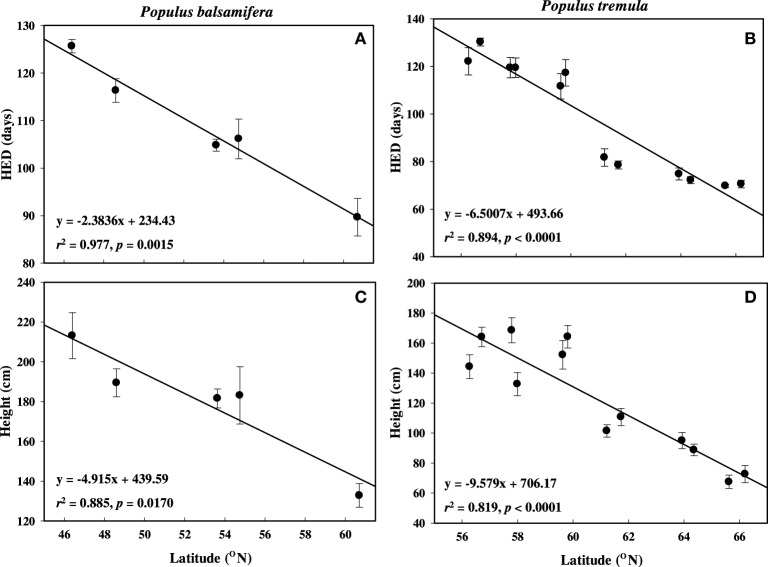
**Mean height elongation duration (HED) and height growth across latitude used in this study**. (A,C) P. balsamifera and (B,D) P. tremula. Error bars are śSE of the means.

In both balsam poplar and aspen, HED and height had strong inverse relationships with latitude (Tables 2, 3, Figure 3). Regardless of provenance, buds flushed within a few days of each other as soon as spring temperatures permitted, whereas dates of bud set spanned a much greater range. For example, in 2007, the White Horse balsam poplar population set bud in the first week of August (X = Julian day 217) whereas the Fredericton population set bud in the first week of September (X = Julian day 247). High-latitude populations set bud early compared to low-latitude populations and population rankings were consistent year-to-year in both common gardens (not shown).

**Figure 4:**
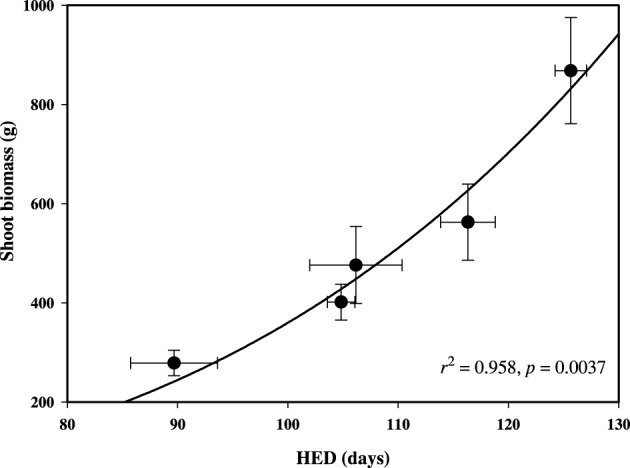
**Relationship between shoot biomass and height elongation duration (HED) among populations of P. balsamifera at the Indian Head common garden (50.33° N, 105.73° W)**. Non-linear regression Equation (2) (*f* = *y*_0_ + *a* ^*^ *x*) is plotted. Error bars ± standard errors of the means.

Balsam poplar shoot biomass was determined at the end of the third growing season and HED was negatively correlated with latitude of origin (Table 2). HED (*r*^2^ = 0.958; *p* = 0.0037) was a better predictor of shoot biomass than GCP (Figure 4, Supplement 2). Because A decreases with HED while biomass increases, biomass and A were negatively correlated (*r* = −0.422, *p* < 0.05). Mean shoot biomass was four-fold greater for the population from Fredericton (low-latitude) than for the population from White Horse (high-latitude) (*r* = −0.904, *p* < 0.001, Table 2). GCP exceeded HED by an average of 67 days, ranging from 50 to 78 days across populations with no clear latitudinal trend.

## Discussion

### Photosynthesis in balsam poplar and aspen

In spite of relatively short photoperiods at the Indian Head common garden, and less so at Sävar, all genotypes went through a period of active growth before they set bud. Photosynthetic rates measured during this period increased with increasing latitude of origin in aspen but not in balsam poplar. The studies by Benowicz et al. (2000), Gornall and Guy (2007), and Soolanayakanahally et al. (2009) suggest that a greater rate of photosynthesis in high-latitude tree genotypes is an adaptation to short growing season length. A trend toward increasing rates of net photosynthesis in relation to increasing latitude of origin has been seen in several plants (Reich et al., 1996), and even lichens (Schipperges et al., 1995). A similar relationship appears to exist with elevation. Ovaska (1988) found that mountain birch populations from high elevations had greater leaf photosynthetic rates per unit area than those from low elevation. Likewise, Oleksyn et al. (1998) reported that high elevation populations of Norway spruce [Picea abies (L.) Karst.] had higher photosynthetic rates than did populations from lower elevations. Higher A during active growth in short-growing season genotypes does not necessarily persist beyond budset. Johnsen et al. (1996) found that black spruce [Picea mariana (Mill) B.S.P.] trees from Yukon (63° N) had higher A than trees from Ontario (45° N) when measured in a 23-year-old common garden during the peak of summer (June–July), but this ranking was reversed when measurements were made later in the season (September–October; i.e., after bud set).

Higher photosynthetic rates in high elevation populations of Norway spruce were supported by a higher percentage of nitrogen in needles with no changes in LMA (Oleksyn et al., 1998). In contrast, in the present study, and under greenhouse conditions (Soolanayakanahally et al., 2009), high latitude populations of balsam poplar tended to have higher photosynthetic rates (*p* < 0.06), as well as significantly higher LMA and leaf N density, but not percent N. Aspen, on the other hand, followed neither of these patterns. Although LMA and Leaf N remained highly correlated across all genotypes, they did not correlate with trends in A. In aspen, higher photosynthetic rates in high latitude populations seemed almost entirely due to higher *g*_s_. Variation in foliar nitrogen among different tree populations had no effect or was inconsistent with photosynthetic rate in Pinus strobus L. (Reich and Schoettle, 1988) and Pinus radiata D. Don. (Sheriff et al., 1986), which also appears to be the case in field-grown aspen. The leaf optical properties, light intensity, and leaf water content could influence CCI values (Biber, 2007), and an intriguing result of greater CCI with latitude while leaf N declines remains largely unexplained in aspen. In addition, the trembling nature of aspen leaves with similar greenness on either side of lamina might scatter the light signals in a different way compared to balsam poplar leaves. Overall, physiological and leaf functional traits may account for variation in growth and biomass; however their expression is strongly controlled by daylength and seasonality (Yu et al., 2001; McKown et al., 2013b).

### Seasonal growth phenology and height

Photosynthetic rates were significantly higher in more northern populations of aspen, and a similar trend was observed in balsam poplar. Although photosynthetic rates tended to be higher in the populations adapted to shorter growing seasons, these populations grew less because of earlier bud set. However, stem height was the best predictor of biomass among fast growing hybrid poplars for use in poplar breeding and tree improvement (Rae et al., 2004). Populations of both species were not at all differentiated in terms of date of bud flush, but there was strong clinal variation in the timing of bud set (Luquez et al., 2007; Soolanayakanahally et al., 2013). In temperate climates, spring temperatures increase and cumulative heat sums have a strong effect on the timing of bud flush (Lechowicz, 1984; Olson et al., 2013). In addition, chilling requirements might also influence bud flush date (Campbell and Sugano, 1975). However, fall bud set is primarily controlled by day length. Pauley and Perry (1954) observed earlier height growth cessation (bud set) in latitudinally diverse populations of Populus species which continued to grow until the daylength fell below a critical threshold that varied with the latitude of origin. The initiation of bud formation is visually apparent within a few days of growth cessation. Our findings and other studies highlight the generality of adaptive clines in bud set (Howe et al., 1995; Li et al., 2003). The period of HED is, therefore, well approximated by the number of days between the recorded dates of bud flush and bud set. When grown outside, the high latitude populations of balsam poplar and aspen accomplished less growth during the summer (Tables 2, 3). Plant height and shoot biomass (Figure 4) were tightly related to HED. In contrast, when height growth cessation was avoided under extended days in a greenhouse, height increment paralleled A in 21 latitudinally different provenances of balsam poplar (Soolanayakanahally et al., 2009). Indeed, short-season genotypes appear to have higher peak rates of stem elongation during free growth, even in outdoor common gardens (pers. comm. Salim Silim). Similarly, Mylecraine et al. (2005) and Mimura and Aitken (2007) found faster spring growth rates among northern populations of Atlantic white-cedar [Chamaecyparis thyoides (L.) B.S.P.] and Sitka spruce, respectively, in range-wide provenance tests encompassing their entire latitudinal ranges (29–44° N and 40–61° N, respectively).

Körner and Renhardt (1987) suggested that high latitude herbaceous perennials invest more in root growth to protect their productivity in case of shoot loss caused by growing season frost events or during winter. It is possible that trees and shrubs may do likewise, reducing potential shoot growth in northern provenances. Latitudinal clines in height growth cessation in response to photoperiod were first documented in European aspen by Sylvén (1940, in Pauley, 1949) and in North American cottonwoods by Pauley and Perry (1954). Photoperiodic responses among black cottonwood ecotypes were also observed by Howe et al. (1995). Cannell and Willett (1976) studied carbon partitioning in potted black cottonwood seedlings from 46 to 58° N, but they reported that all provenances had similar carbon partitioning between roots and shoots until height growth cessation. However, because carbon partitioning after bud set favors roots (Ledig et al., 1970), which continue to grow, black cottonwood from northern provenances finished the growing season with a higher root:shoot ratio. Enhanced late-season partitioning to root growth, compounded over years, might increase belowground respiratory costs to further impact relative rates of shoot growth. Cannell and Willett (1976), however, reported that differences in root:shoot ratio did not persist year-to-year because they were corrected each spring by proportionally greater shoot growth among northern genotypes.

### Comparative physiology of populus

For comparative purposes, Table 4 summarizes correlations reported here in outdoor common gardens for P. balsamifera and P. tremula, as well as for P. trichocarpa from Gornall and Guy (2007) and McKown et al. (2013a). The table also presents data for P. balsamifera grown under extended photoperiod in an indoor “common garden” as reported by Soolanayakanahally et al. (2009).

**Table 4:** **Comparative physiology among P. balsamifera, P. tremula, and P. trichocarpa**.

**Variables**	*P. balsamifera*		***P. tremula***	***P. trichocarpa***	
	**Greenhouse (Soolanayakanahally et al., 2009) *n* = 210**	**Field (present study) *n* = 30**	**Field (present study) *n* = 116**	**Field (Gornall and Guy, 2007) *n* = 30**	**Field (McKown et al., 2013a) *n* = 461**
A	+^***^	ns	+^***^	+^*^	+^***^
*g*_s_	ns	+^*^	+^***^	+^*^	+^*^
WUE_i_	+^***^	−^*^	ns	ns	−^*^
δ^13^C	+^*^	ns	ns	ns	ns
Leaf N	+^***^	+^***^	−^***^	+^*^	+^*^
CCI	+^***^	+^*^	+^***^	not available	+^*^
Height	+^***^	−^*^	−^***^	−^*^	−^*^
LMA	+^***^	+^***^	ns	ns	ns

*Summary of correlations for all genotypes between physiological and morphological variables with latitude. All results are from present study except where indicated*.

* *p < 0.05*;

**p < 0.01;

*** *p < 0.001; ns, no significant change*.

*The statistical tests for the present study are from Tables 2, 3. A, assimilation rate (μmol CO_2_ m^−2^ s^−1^); g_s_, stomatal conductance (mol H_2_O m^−2^ s^−1^); WUE_i_, intrinsic water use efficiency (μmol CO_2_ mol^−1^ H_2_O); δ^13^C, carbon isotope composition of leaf (‰); Leaf N, leaf nitrogen density (μmol N cm^−2^); CCI, chlorophyll content index; Height (cm); LMA, leaf mass area (mg cm^−2^)*.

Across all studies, photosynthetic assimilation rates (A) were always measured during active growth irrespective of the growth conditions, and in all five studies, A increased with latitude of origin (field grown balsam poplar, *p* > 0.05). The consistency of this geographic cline in allopatric Populus species, as well as other woody plants, is strongly suggestive of its global adaptive significance. We have previously noted that higher A in populations from higher latitudes can be achieved in different ways. For example, Soolanayakanahally et al. (2009) found that higher internal (mesophyll) conductance, and not *g*_s_, was associated with enhanced A in greenhouse-grown P. balsamifera, whereas *g*_s_ was correlated with A across field-grown P. trichocarpa populations (*r* = 0.757, *p* < 0.05, Gornall and Guy, 2007; *r* = 0.70, *p* < 0.05, McKown et al., 2013a). Mesophyll conductance was not estimated in both studies, but *g*_s_ increased with latitude in outdoor common gardens across all three species considered in Table 4, including P. balsamifera. Different trends in *g*_s_ between the field-grown and greenhouse-grown balsam poplars largely account for different trends in WUE_i_, which decreased with latitude of origin in the former but increased in the latter. However, WUE_*i*_ was not significant with latitude of origin in field-grown P. termula (Table 4). No latitudinal pattern in WUE_i_ was observed in field-grown black cottonwood by Gornall and Guy (2007), but in a broader study involving more populations, WUE_i_ decreased with latitude also in this species (McKown et al., 2013a).

Trends in leaf δ^13^C were in full concurrence with WUE_i_. A strong correlation between δ^13^C and WUE_i_ has been reported for numerous tree species (Zhang and Marshall, 1994; Zhang et al., 2004; Pointeau and Guy, 2014). Conversely, the relationship between δ^13^C and WUE_i_ at the leaf level did not scale up to harvestable stems in Eucalyptus grandis (Olbrich et al., 1993); hence, recurrent measurements of gas exchange across the growing season are crucial to better infer the outcomes (Wang et al., 2013). The differences in δ^13^C values among the P. balsamifera and P. tremula populations were small and non-significant with latitude of origin (Tables 2, 3). In P. balsamifera, however, the cline was positive under greenhouse conditions (Soolanayakanahally et al., 2009), but trended in the opposite direction in the outdoor common garden (this study). Several authors have described in situ variation in δ^13^C with latitude and elevation within species (Zhang et al., 1993). Such variation may be genetic or environmental or both. Clearly, however, growth in a common garden to assess the genetic component will not necessarily exclude the effects of environment. As noted above, earlier height growth cessation in high latitude genotypes may favor increased investment into root growth and a higher root:shoot ratio. Increased partitioning to roots might permit access to water from a greater relative volume of soil resulting in higher *g*_s_, lower WUE_i_, and increased isotope discrimination. When photoperiodic restrictions are removed under greenhouse conditions, high latitude genotypes can exhibit greater height growth than low-latitude genotypes. This reversal in height rankings among trees grown in the greenhouse (long photoperiod) vs. an outdoor common garden (short photoperiod) complicates the interpretation of the adaptive significance of water-use efficiency and the evaluation of growth rates for tree improvement. Hence, provenance trial results should be interpreted cautiously if not replicated at different latitudes.

Differences in LMA and Leaf N accounted for most of the variation in A reported by Soolanayakanahally et al. (2009) for balsam poplar populations under greenhouse conditions and, in the present study, an outdoor common garden. Leaf N density, but not LMA, was also associated with enhanced A in black cottonwood (Gornall and Guy, 2007; McKown et al., 2013a). However, aspen showed no association between A and Leaf N or between A and LMA (Supplement 1). CCI, on the other hand, paralleled A across all studies where it was assessed. This observed variance in leaf functional traits seems to be largely driven by the adaptation of a given population to latitude (daylength); however, common garden environmental factors might also influence trait-to-trait relationships. Other studies also found weak correlations between greenhouse juvenile and field mature trees (Cornelissen et al., 2003; Smith et al., 2011). As expected, final height was negatively correlated with latitude of origin across all three Populus species when grown outdoors under a natural photoperiodic regime.

## Conclusions

Latitudinal clines in A exist among allopatric Populus species adapted to similar environments on different continents. We interpret this as a common and, therefore, an important adaptation to short growing seasons. In fact, this appears to be a convergent trait in that the underlying physiological mechanisms responsible for higher A are not consistent across species. In spite of intrinsically higher photosynthetic rates, high latitude populations consistently accomplish less height during the growing season as a result of earlier bud set leading to growth cessation. A shortcoming of unreplicated outdoor common gardens or provenance trails to assess growth rates for purposes of tree improvement is the crucial role of photoperiod in limiting plant height (Howe et al., 1995; Way and Montgomery, 2014). Because height growth is largely predetermined by phenological events, the effects of other important traits that determine performance are effectively obscured. Furthermore, subsequent effects on growth complicate the interpretation of other important adaptive traits such as root:shoot ratio, WUE_i_, and δ^13^C (McKown et al., 2013b).

## Author contributions

All authors designed and performed the experiments.

## Conflict of interest statement

The authors declare that the research was conducted in the absence of any commercial or financial relationships that could be construed as a potential conflict of interest.
