# Supplementary material for: Comparative physiology of allopatric Populus species: geographic clines in photosynthesis, height growth, and carbon isotope discrimination in common gardens
Source: Front Plant Sci. 2015 Jul 14;6:528. doi: 10.3389/fpls.2015.00528 (PMC4500902; doi:10.3389/fpls.2015.00528)
Supplement: Supplementary file 1 [file DataSheet1.DOCX]

***Supplementary Material***

**Comparative physiology of allopatric *Populus* species: Geographic clines in photosynthesis, height growth and carbon isotope discrimination in common gardens**

Raju Y. Soolanayakanahally^1,2*^, Robert D. Guy^2^, Nathaniel R. Street^3^, Kathryn M. Robinson^3^, Salim N. Silim^1^, Benedicte R. Albrectsen^3^ and Stefan Jansson^3^

^1^ Agroforestry Development Centre, Agriculture and Agri-Food Canada, Indian Head, SK, Canada

^2^ Department of Forest and Conservation Sciences, University of British Columbia, Vancouver, BC, Canada

^3^ Department of Plant Physiology, Umeå Plant Science Centre, Umeå University, Umeå, Sweden

***Correspondence:**

Raju Soolanayakanahally

Agroforestry Development Centre

Agriculture and Agri-Food Canada

#2 Government Road

Indian Head, SK, S0G 2K0, Canada

[raju.soolanayakanahally@agr.gc.ca](mailto:raju.soolanayakanahally@agr.gc.ca)

**Supplement 1**. Correlation coefficients among physiological variables. Significant correlations are indicated in bold (*p* < 0.05), and correlations that were significant after Bonferroni correction are indicated with an asterisk (*p* < 0.0024).

| ***P. balsamifera*** (n = 30) | *g*_s_ | WUE_i_ | δ^13^C | Leaf N | CCI | LMA |
| --- | --- | --- | --- | --- | --- | --- |
| *A* | **0.816*** | **-0.505*** | **-0.439** | **0.398** | 0.328 | **0.407** |
| *g*_s_ | 1 | **-0.904*** | **-0.523*** | 0.352 | **0.438** | **0.453** |
| WUE_i_ |  | 1 | **0.446** | -0.243 | **-0.369** | **-0.381** |
| δ^13^C |  |  | 1 | -0.060 | -0.250 | -0.158 |
| Leaf N |  |  |  | 1 | **0.335** | **0.902*** |
| CCI |  |  |  |  | 1 | **0.545*** |
|  | | | | | | |
| ***P. tremula*** (n = 116) | *g*_s_ | WUE_i_ | δ^13^C | Leaf N | CCI | LMA |
| *A* | **0.717*** | **-0.337*** | -0.168 | 0.008 | **0.553*** | 0.109 |
| *g*_s_ | 1 | **-0.809*** | -0.158 | 0.080 | **0.366*** | **0.206** |
| WUE_i_ |  | 1 | 0.154 | -0.070 | -0.103 | **-0.195** |
| δ^13^C |  |  | 1 | **0.263** | -0.058 | 0.006 |
| Leaf N |  |  |  | 1 | 0.056 | **0.824*** |
| CCI |  |  |  |  | 1 | 0.097 |

*A*, assimilation rate (μmol CO_2_ m^-2^ s^-1^); *g*_s_, stomatal conductance (mol H_2_O m^-2^ s^-1^); WUE_i_, intrinsic water use efficiency (μmol CO_2_ mol^-1^ H_2_O); δ^13^C, carbon isotope composition of leaf (‰); Leaf N, leaf nitrogen density (μmol N cm^-2^); CCI, chlorophyll content index; LMA, leaf mass area (mg cm^-2^).

**Supplement 2**. Correlation coefficients among growth variables for *P. balsamifera*. Significant correlations are indicated in bold (*p* < 0.05), and correlations that were significant after Bonferroni correction are indicated with an asterisk (*p* < 0.0083).

| Biomass variables (n = 30) | HED | Height | Biomass |
| --- | --- | --- | --- |
| GCP | **0.729*** | **0.723*** | **0.532*** |
| HED | 1 | **0.775*** | **0.759*** |
| Height |  | 1 | **0.727*** |

GCP, green cover period (days); HED, height elongation duration (days); Height (cm); Biomass (g)
